# Cysteine Peptidase B Regulates *Leishmania mexicana* Virulence through the Modulation of GP63 Expression

**DOI:** 10.1371/journal.ppat.1005658

**Published:** 2016-05-18

**Authors:** Pierre-André Casgrain, Caroline Martel, W. Robert McMaster, Jeremy C. Mottram, Martin Olivier, Albert Descoteaux

**Affiliations:** 1 INRS- Institut Armand-Frappier and the Center for Host-Parasite Interactions, Laval, Canada; 2 The Research Institute of the McGill University Health Centre, Montréal, Canada; 3 Immunity and Infection Research Centre, Vancouver Coastal Health Research Institute, Department of Medical Genetics, University of British Columbia, Vancouver, Canada; 4 Centre for Immunology and Infection, Department of Biology, University of York, Wentworth Way Heslington, York, United Kingdom; University of Texas Medical Branch, UNITED STATES

## Abstract

Cysteine peptidases play a central role in the biology of *Leishmania*. In this work, we sought to further elucidate the mechanism(s) by which the cysteine peptidase CPB contributes to *L*. *mexicana* virulence and whether CPB participates in the formation of large communal parasitophorous vacuoles induced by these parasites. We initially examined the impact of *L*. *mexicana* infection on the trafficking of VAMP3 and VAMP8, two endocytic SNARE proteins associated with phagolysosome biogenesis and function. Using a CPB-deficient mutant, we found that both VAMP3 and VAMP8 were down-modulated in a CPB-dependent manner. We also discovered that expression of the virulence-associated GPI-anchored metalloprotease GP63 was inhibited in the absence of CPB. Expression of GP63 in the CPB-deficient mutant was sufficient to down-modulate VAMP3 and VAMP8. Similarly, episomal expression of GP63 enabled the CPB-deficient mutant to establish infection in macrophages, induce the formation of large communal parasitophorous vacuoles, and cause lesions in mice. These findings implicate CPB in the regulation of GP63 expression and provide evidence that both GP63 and CPB are key virulence factors in *L*. *mexicana*.

## Author Summary

The parasite *Leishmania mexicana* expresses several cysteine peptidases of the papain family that are involved in processes such as virulence and evasion of host immune responses. The cysteine peptidase CPB is required for survival within macrophages and for lesion formation in susceptible mice. Upon their internalization by macrophages, parasites of the *L*. *mexicana* complex induce the formation of large communal parasitophorous vacuoles in which they replicate, and expansion of those large vacuoles correlates with the ability of the parasites to survive inside macrophages. Here, we found that CPB contributes to *L*. *mexicana* virulence (macrophage survival, formation and expansion of communal parasitophorous vacuoles, lesion formation in mice) through the regulation of the virulence factor GP63, a *Leishmania* zinc-metalloprotease that acts by cleaving key host cell proteins. This work thus elucidates a novel *Leishmania* virulence regulatory mechanism whereby CPB controls the expression of GP63.

## Introduction

The protozoan *Leishmania* parasitizes macrophages and causes a spectrum of human diseases ranging from self-healing cutaneous lesions to a progressive visceral infection that can be fatal if left untreated. Infection is initiated when promastigote forms of the parasite are inoculated into the mammalian host by infected sand flies and are internalized by phagocytes. Inside macrophages, promastigotes differentiate into amastigotes to replicate within phagolysosomal compartments also known as parasitophorous vacuoles (PVs). Upon their internalization, *L*. *donovani* and *L*. *major* promastigotes arrest phagolysosomal biogenesis and create an intracellular niche favorable to the establishment of infection and to the evasion of the immune system [1, 2]. Disruption of the macrophage membrane fusion machinery through the action of virulence factors plays an critical role in this PV remodeling. Hence, insertion of the promastigote surface glycolipid lipophosphoglycan (LPG) into the PV membrane destabilizes lipid microdomains and causes exclusion of the membrane fusion regulator synaptotagmin V from the PV [2–4]. Similarly, the parasite GPI-anchored metalloprotease GP63 [5, 6] redistributes within the infected cells and cleaves key Soluble NSF Attachment Protein Receptors (SNAREs) and synaptotagmins to impair phagosome functions [1, 7].

Whereas *L*. *major* and *L*. *donovani* multiply in tight individual PVs, parasites of the *L*. *mexicana* complex (*L*. *mexicana*, *L*. *amazonensis*) replicate within large communal PVs. Relatively little is known about the host and parasite factors involved in the biogenesis and expansion of those communal PVs. Studies with *L*. *amazonensis* revealed that phagosomes containing promastigotes fuse extensively with late endosomes/lysosomes within 30 minutes post-infection [8]. At that stage, parasites are located within small individual compartments and by 18 to 24 hours large PVs containing several parasites are observed. The rapid increase in the size of those PVs requires extensive fusion with secondary lysosomes and correlates with the depletion of those organelles in infected cells [9–11]. Homotypic fusion between *L*. *amazonensis*-containing PVs also occurs, but its contribution to PV enlargement remains to be further investigated [12]. These studies highlighted the contribution of the host cell membrane fusion machinery in the biogenesis and expansion of large communal PVs and are consistent with a role for endocytic SNAREs in this process [13]. Interestingly, communal PVs interact with the host cell’s endoplasmic reticulum (ER) and disruption of the fusion machinery associated with the ER and Golgi inhibits parasite replication and PV enlargement [14–16].

The *Leishmania*-derived molecules involved in the expansion of the communal PVs remains to be identified. LPG and other phosphoglycans do not play a significant role in *L*. *mexicana* promastigote virulence and PV formation [17], in contrast to *L*. *major* and *L*. *donovani* [2]. Cysteine peptidases (CP) are a large family of papain-like enzymes that play important roles in the biology of *Leishmania* [18]. Three members of these papain-like proteases are expressed by *L*. *mexicana* and the generation of CP-deficient mutants revealed that CPB contributes to the ability to infect macrophages and to induce lesions in BALB/c mice [19–21]. The precise mechanism(s) by which CPB participates in the virulence of *L*. *mexicana* is poorly understood. Previous studies revealed that CPB traffics within and outside infected macrophages [18]. In infected macrophages, CPB alters signal transduction and gene expression through the activation of the protein tyrosine phosphatase PTP-1B and the cleavage of transcription factors responsible for the expression of genes involved in host defense and immunity [20, 22]. The observation that CPs interfere with the host immune response through the degradation of MHC class II molecules and invariant chains present in PVs housing *L*. *amazonensis* [23], raises the possibility that CPB participates in the modulation of PV composition and function.

In this study, we sought to gain insight into the mechanism by which CPB contributes to *L*. *mexicana* virulence, with a focus on the PV. We provide evidence that CPB participates in PV biogenesis and virulence through the regulation of GP63 expression.

## Results

### CPB enables *L*. *mexicana* to down-modulate VAMP3 and VAMP8

**Fig 1:**
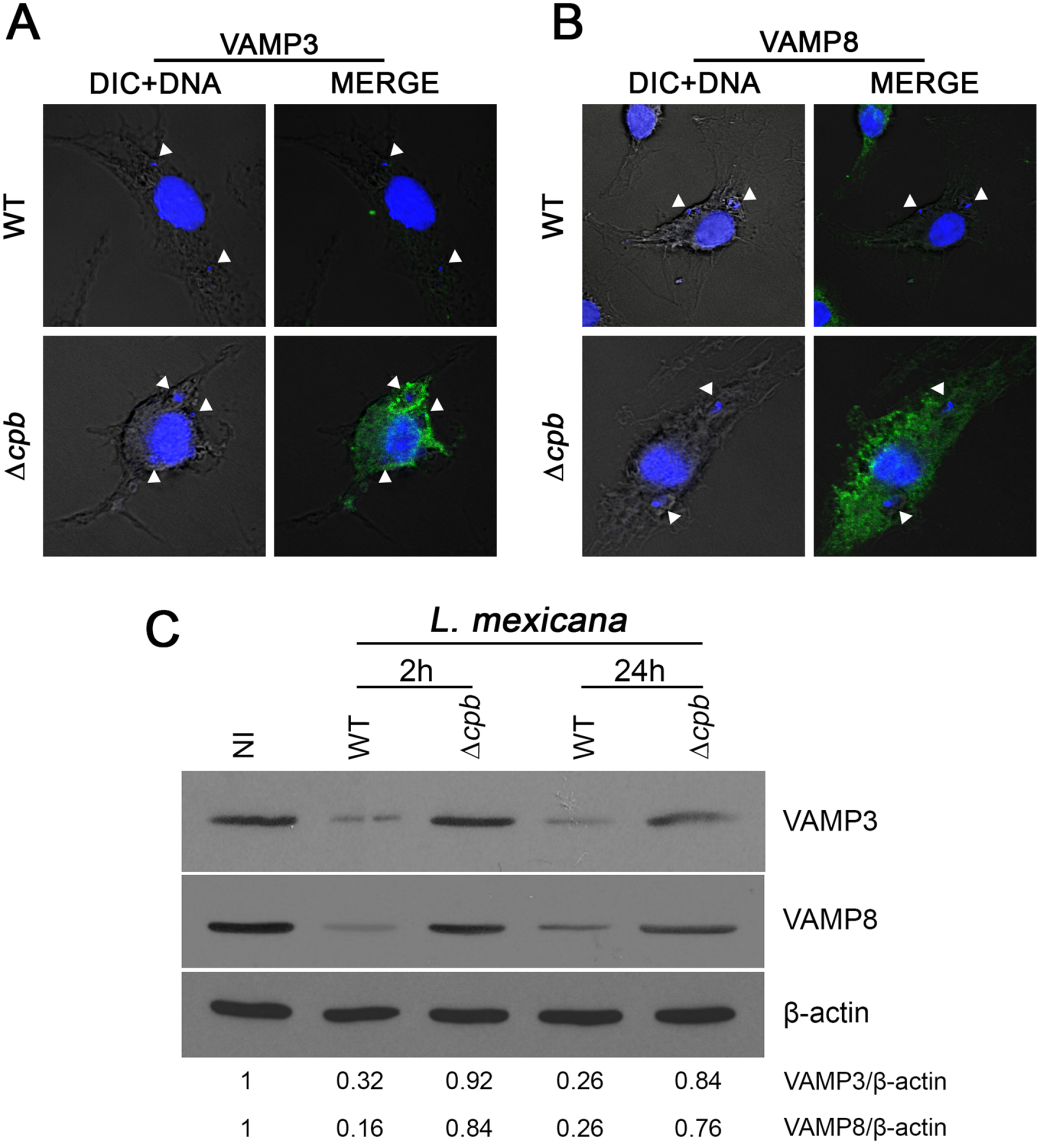
Down-modulation of VAMP3 and VAMP8 by *L*. *mexicana*. BMM were infected with serum-opsonized stationary phase *L*. *mexicana* (WT and *Δcpb*) promastigotes for 2 h. VAMP3 (**A**) and VAMP8 (**B**) levels (green) were then visualized by confocal microscopy. Macrophage and parasite nuclei are shown in blue (DAPI). Internalized parasites are denoted by white arrowheads. In **(C)**, VAMP3 and VAMP8 levels in total cell extracts were assessed by Western blot analysis. Each immunofluorescence assay was done on 300 phagosomes on triplicate coverslips in two independent experiments and Western blot analyses were performed twice in three independent experiments. VAMP3 and VAMP8/β-actin ratios were determined by densitometry. Original magnification X63.

**Fig 2:**
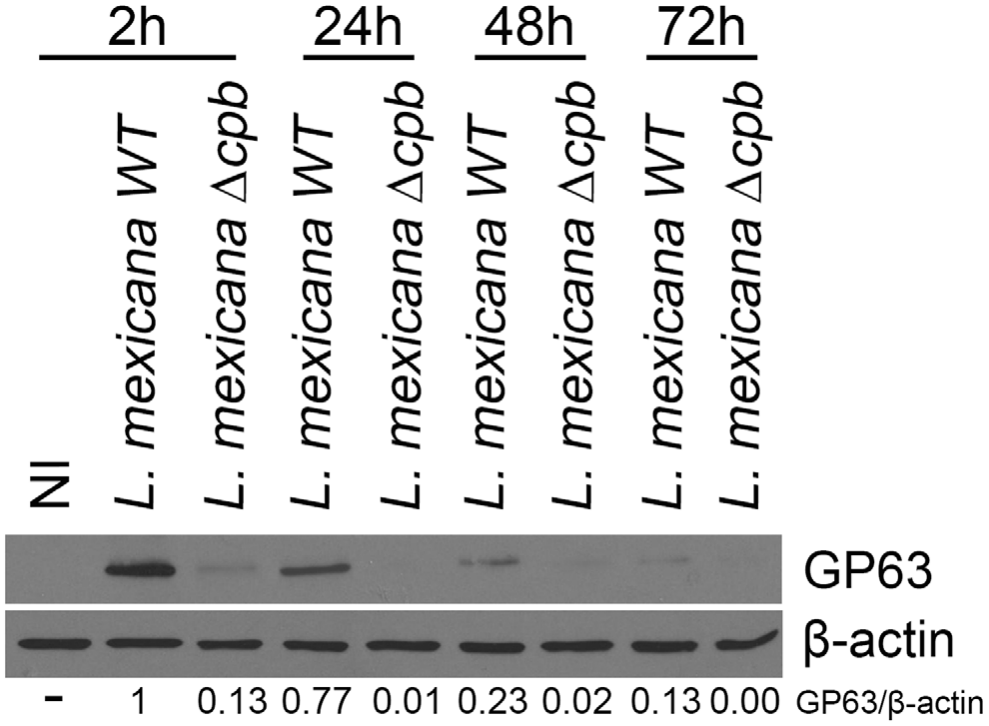
GP63 is down-modulated in the *L*. *mexicana* Δ*cpb* mutant. BMM were infected with serum-opsonized stationary phase *L*. *mexicana* (WT and *Δcpb*) promastigotes for 2 h, 24 h, 48 h and 72 h. Total cell extracts were assayed for GP63 levels by Western blot analysis. GP63/ β-actin ratios were determined by densitometry. Similar results were obtained in three independent experiments.

Formation and expansion of communal PVs hosting *L*. *mexicana* involve fusion between PVs and endocytic organelles, as well as homotypic fusion among PVs [10–12]. To identify the host and parasite factors involved in this process, we embarked on a study to elucidate the fate of endosomal SNAREs during infection of macrophages with *L*. *mexicana*. Given the requirement of CPB for *L*. *mexicana* to replicate normally inside macrophages [19], we included a *L*. *mexicana* CPB-deficient mutant (Δ*cpb*) in our investigation. We infected BMM with either WT or Δ*cpb L*. *mexicana* promastigotes for 2 h and we assessed the distribution of the endosomal SNAREs VAMP3 and VAMP8 by confocal immunofluorescence microscopy. As previously observed during infection with *L*. *major* promastigotes [1], we found a notable reduction in the staining intensity for both VAMP3 (Fig 1A) and VAMP8 (Fig 1B) in BMM infected with WT *L*. *mexicana*, but this was not observed with Δ*cpb*. This reduction in staining intensity correlated with a down-modulation of VAMP3 and VAMP8 proteins in BMM infected with WT *L*. *mexicana*, compared to cells infected with Δ*cpb* (Fig 1C). These results suggested that *L*. *mexicana* causes the reduction of VAMP3 and VAMP8 levels in infected BMM through the action of CPB. However, we considered the possibility that CPB acted indirectly on VAMP3 and VAMP8 because we previously found that GP63 targets those SNAREs in *L*. *major*-infected BMM [1]. We therefore ensured that similar levels of GP63 were present in lysates of BMM infected with WT and Δ*cpb L*. *mexicana* promastigotes. As shown in Fig 2, GP63 was detected in lysates of BMM infected with WT *L*. *mexicana* up to 72 h post-infection, when the parasites replicate as amastigotes. The important reduction in GP63 levels at this time point is consistent with previously published data showing a 90% reduction in the amount of GP63 detected in amastigotes with respect to promastigotes [24, 25]. Surprisingly, we found that GP63 was barely detectable in BMM infected with Δ*cpb* at all time points tested. This observation raised the possibility that the lack of VAMP3 and VAMP8 down-regulation in Δ*cpb*-infected BMM was due to defective expression of GP63.

### CPB is required for GP63 expression

**Fig 3:**
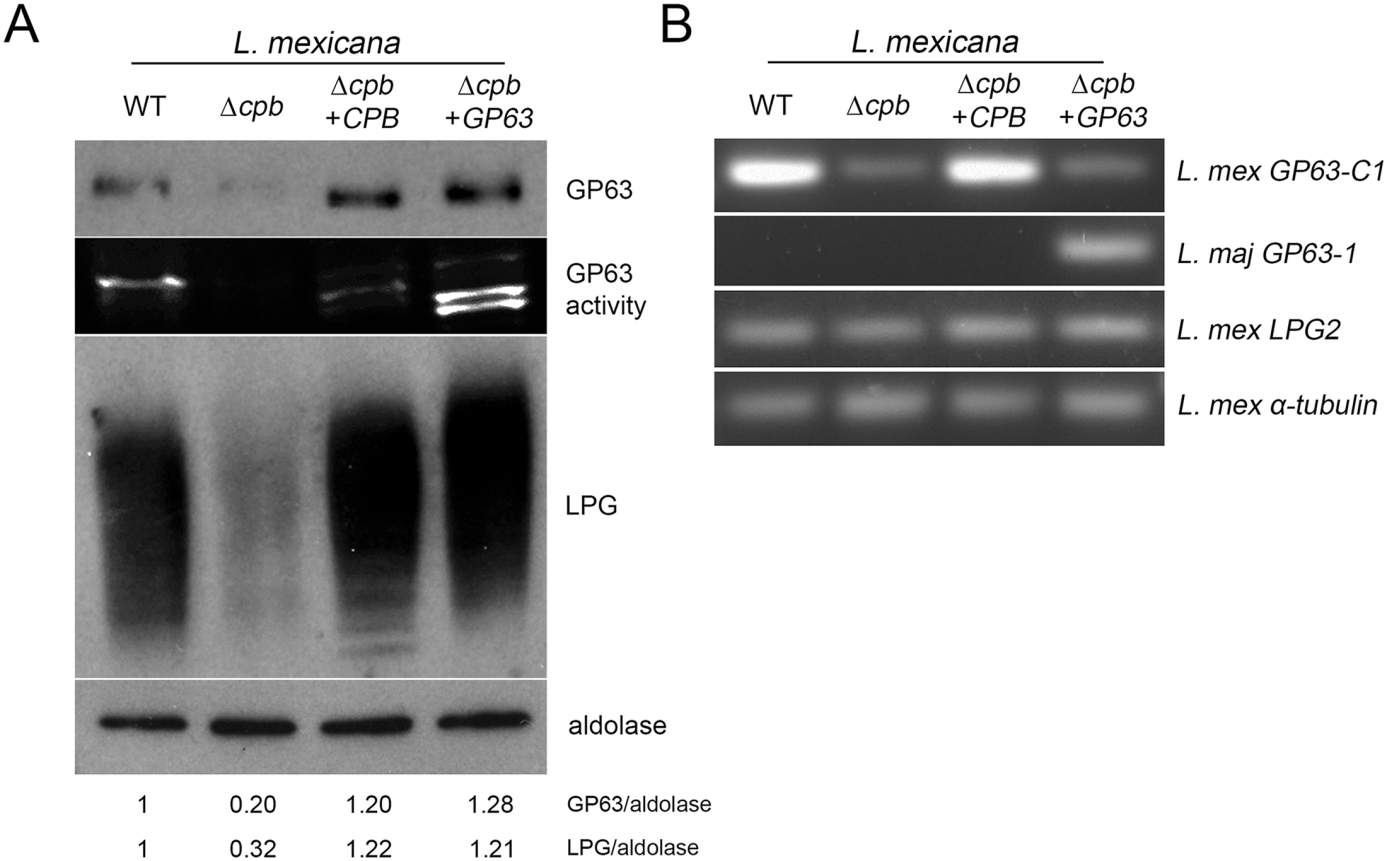
Expression of GP63 and LPG is impaired in the absence of CPB. **(A)** Stationary phase promastigotes were lysed and total cell extracts were analysed by Western blotting and zymography for GP63 levels and activity and for LPG levels. Aldolase was used as a loading control. GP63 and LPG/aldolase ratios were determined by densitometry. **(B)** Promastigote total RNA was extracted and reverse transcription followed by PCR was performed to assess mRNA levels for *L*. *mexicana GP63-C1*, *LPG2*, and α-tubulin, and *L*. *major GP63-1*. Similar results were obtained in three independent experiments.

To address the issue of GP63 down-regulation in *L*. *mexicana* Δ*cpb*, we first determined whether complementation of Δ*cpb* with the *CPB* gene array (Δ*cpb+CPB*) restores wild type GP63 levels. As shown in Fig 3A, GP63 levels and activity are down-modulated in the Δ*cpb* mutant, and complementation with the *CPB* array restored GP63 levels and activity similar to those observed in WT parasites. It was previously reported that expression of the cell surface glycolipid LPG and of GP63 may share common biosynthetic steps [26–29]. We therefore evaluated the levels of LPG in lysates of WT, Δ*cpb*, Δ*cpb*+*CPB*, and Δ*cpb*+*GP63* parasites by Western blot analysis. Strikingly, similar to GP63, LPG levels were also down-modulated in the *Δcpb* mutant and complementation with either the *CPB* array or *GP63* restored wild type LPG levels. To further investigate the possible role of CPB in the regulation of GP63 expression, we determined the levels of *GP63* mRNA in WT, Δ*cpb*, Δ*cpb*+*CPB*, and Δ*cpb*+*GP63* parasites by RT-PCR. As shown in Fig 3B, *GP63* mRNA levels were highly down-regulated in *Δcpb* and complementation with the CPB array restored wild type levels of GP63 mRNA. Interestingly, complementation of *Δcpb* with *L*. *major GP63* did not increase endogenous *GP63* mRNAs. RT-PCR using *L*. *major* GP63-specific primers showed that this gene is expressed only in Δ*cpb*+*GP63*. Together, these results suggest that CPB controls GP63 mRNA levels at the post-transcriptional level. Clearly, additional studies will be required to elucidate the underlying mechanism(s). Our results also raised the possibility that down-modulation of GP63 in the Δ*cpb* mutant may have accounted for the inability of Δ*cpb* to down-regulate VAMP3 and VAMP8. The finding that expression of GP63 in Δ*cpb* restored LPG levels was unexpected and suggested a role for GP63 in the expression of LPG in *L*. *mexicana*. As it is estimated that at least 25 genes are required for the synthesis, assembly, and transport of the various components of LPG [30], it may be difficult to determine whether GP63 acts on the expression of a LPG biosynthetic gene or on a biosynthetic step. Assessment of *LPG2* gene expression revealed that it was equally expressed WT, Δ*cpb*, Δ*cpb*+*CPB*, and Δ*cpb*+*GP63* parasites. Further studies will be necessary to understand how GP63 expression restores LPG synthesis in Δ*cpb*. Since LPG does not play a major role in the virulence of *L*. *mexicana* [17], the Δ*cpb* mutant expressing exogenous GP63 provides a unique opportunity to address the impact of GP63 on SNARE cleavage, as well as on the *in vitro* and *in vivo* virulence of *L*. *mexicana*.

### GP63 is responsible for the cleavage of VAMP3 and VAMP8 by *L*. *mexicana*

**Fig 4:**
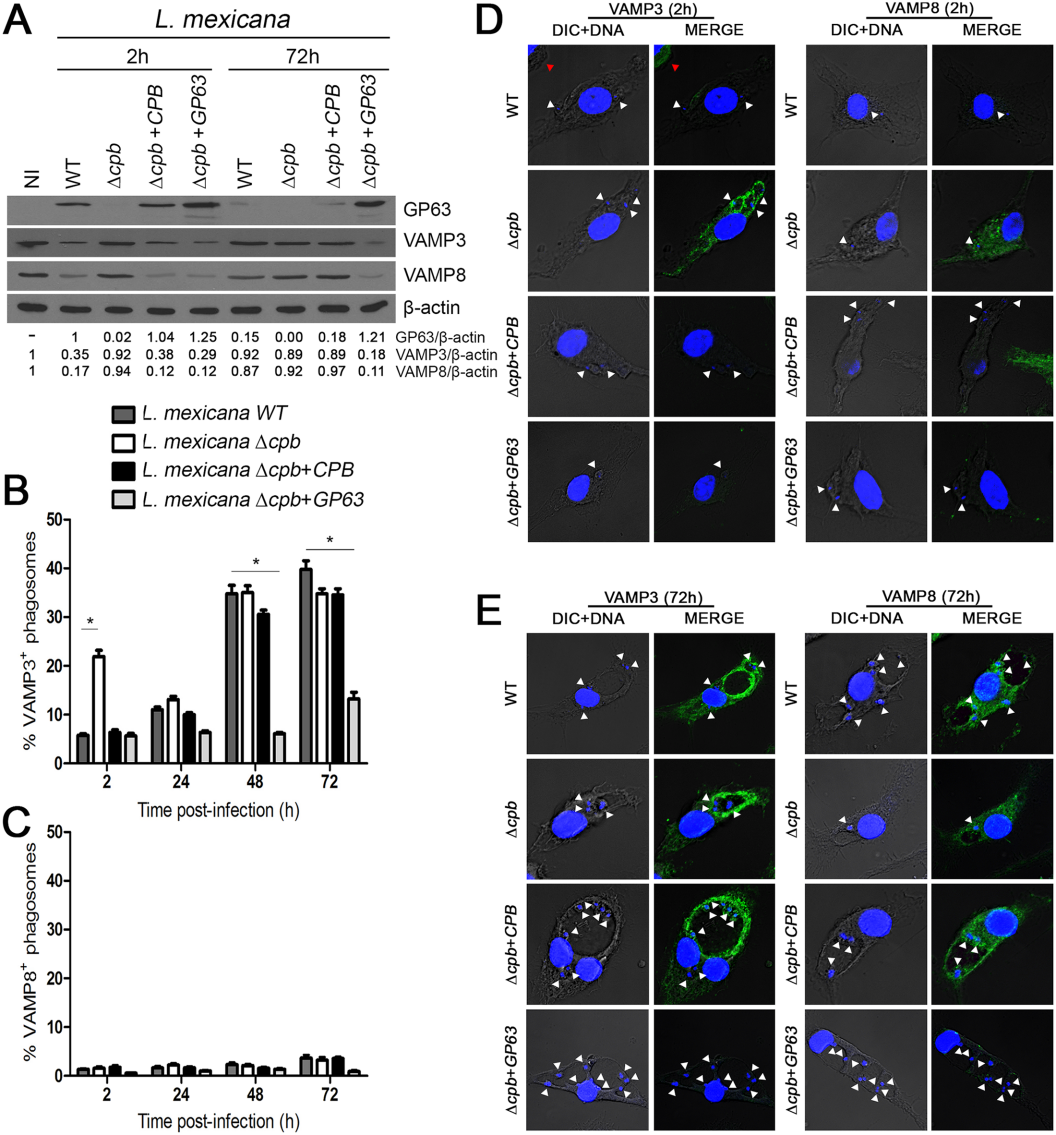
GP63 is responsible for the down-modulation of VAMP3 and VAMP8 in *L*. *mexicana*-infected macrophages. BMM were infected with serum-opsonized stationary phase *L*. *mexicana* (WT, Δ*cpb*, Δ*cpb*+*CPB* and Δ*cpb*+*GP63*) promastigotes for 2 h and 72 h. Total cell extracts were analysed by Western blot **(A).** Similar results were obtained in three independent experiments. VAMP3 and VAMP8 recruitment to the phagosome was visualized by immunofluorescence microscopy and quantified for 300 phagosomes on triplicate coverslips **(B and C)**. Representative pictures from each condition are shown **(D and E)**. Immunofluorescence assays were performed on 300 phagosomes on triplicate coverslips for three independent experiments. *p<0.0001. Original magnification X63.

We next assessed the impact of GP63 on VAMP3 and VAMP8 during *L*. *mexicana* infection. To this end, we infected BMM with either WT, Δ*cpb*, Δ*cpb*+*CPB*, or Δ*cpb*+*GP63 L*. *mexicana* promastigotes for various time points, and we assessed VAMP3 and VAMP8 levels and intracellular distribution. Fig 4A shows that GP63 is present at high levels in lysates of BMM infected for 2 h with WT, Δ*cpb*+*CPB*, and Δ*cpb*+*GP63* promastigotes (compared to lysates of BMM infected with Δ*cpb*). At 72 h post-infection, GP63 levels are strongly reduced in BMM infected with WT and *Δcpb*+*CPB*, whereas they remain elevated in BMM infected with the Δ*cpb*+*GP63* (Fig 4A) [25]. The high levels of GP63 present in BMM infected with Δ*cpb*+*GP63* for 72 h may be related to the fact that expression of the *L*. *major* GP63 gene from the pLEXNeo episomal vector [31] is not under the control of endogenous *GP63* 3' untranslated regions. Western blot analyses revealed that down-regulation of VAMP3 and VAMP8 correlated with GP63 levels expressed by the parasites. Consistently, the staining intensity of VAMP3 and VAMP8 was reduced in BMM infected with GP63-expressing parasites, as assessed by confocal immunofluorescence microscopy (Fig 4D and 4E). These results suggest that GP63 is responsible for the down-modulation of the endosomal SNAREs VAMP3 and VAMP8 in *L*. *mexicana*-infected BMM. Interestingly, we observed recruitment of VAMP3 to PVs containing *L*. *mexicana* parasites at later time points, when promastigotes have differentiated into amastigotes, with the exception of Δ*cpb+GP63 L*. *mexicana* promastigotes (Fig 4B). In contrast, we found that VAMP8 is excluded from *L*. *mexicana*-containing PVs both at early and later time points post-infection, independently of GP63 levels, suggesting that additional mechanisms regulate VAMP8 recruitment to *L*. *mexicana* PVs.

### GP63 expression restores virulence of *Δcpb*

**Fig 5:**
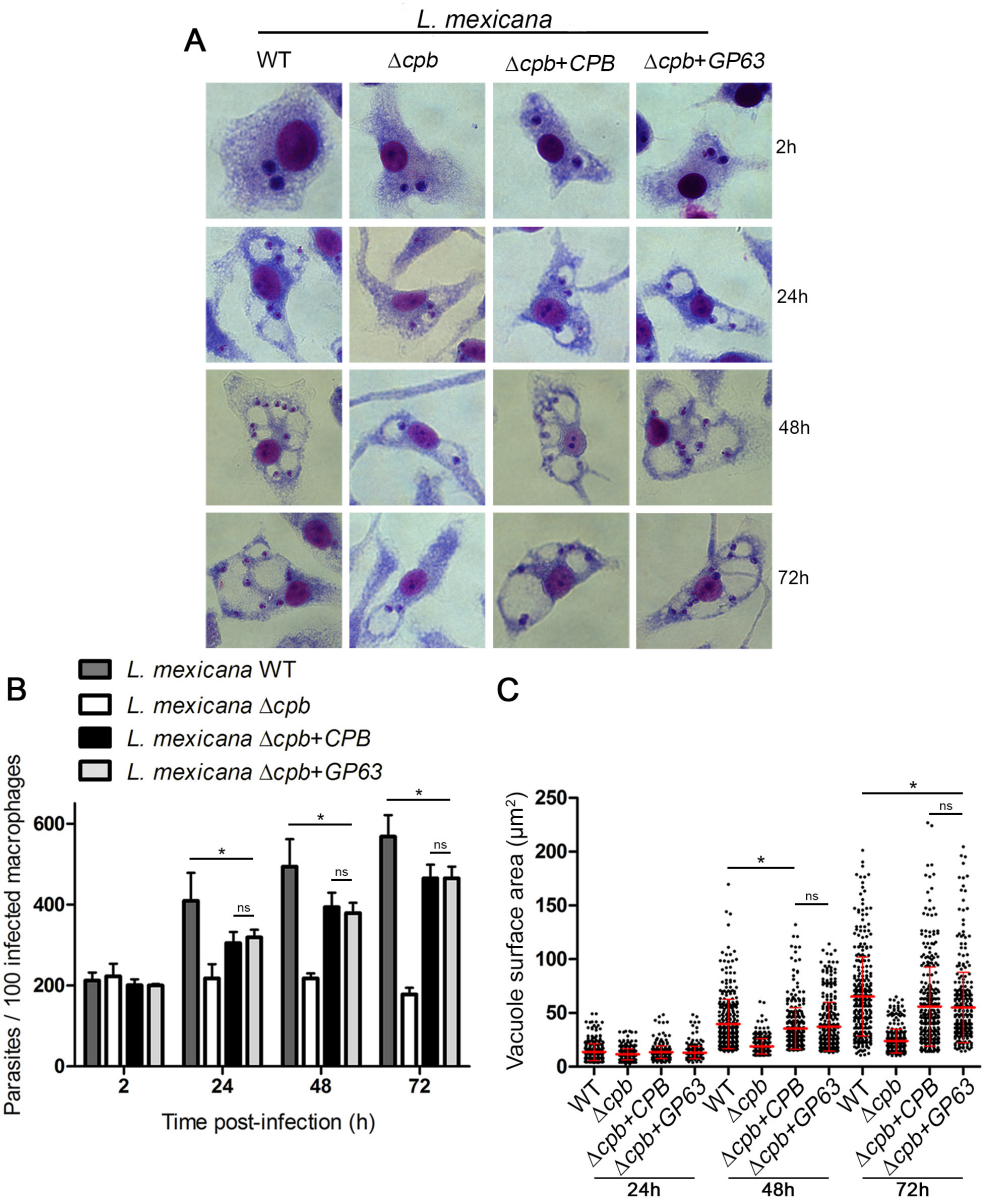
GP63 enables *L*. *mexicana* Δ*cpb* to infect macrophages and induce large PVs. BMM were infected with stationary phase serum-opsonized *L*. *mexicana* (WT, Δ*cpb*, Δ*cpb*+*cpb* and Δ*cpb*+*GP63*) promastigotes for 2 h, 24 h, 48 h and 72 h. Macrophages were stained with the HEMA 3 kit. Representative pictures from each condition are shown **(A)** Parasites were counted in 300 macrophages on triplicate coverslips **(B).** Macrophages were stained with the LAMP1 antibody and vacuole sizes were measured with the ZEN 2012 software **(C)**. Parasitemia and vacuole size was determined on 300 phagosomes in triplicate in three independent experiments. *p<0.0001.

**Fig 6:**
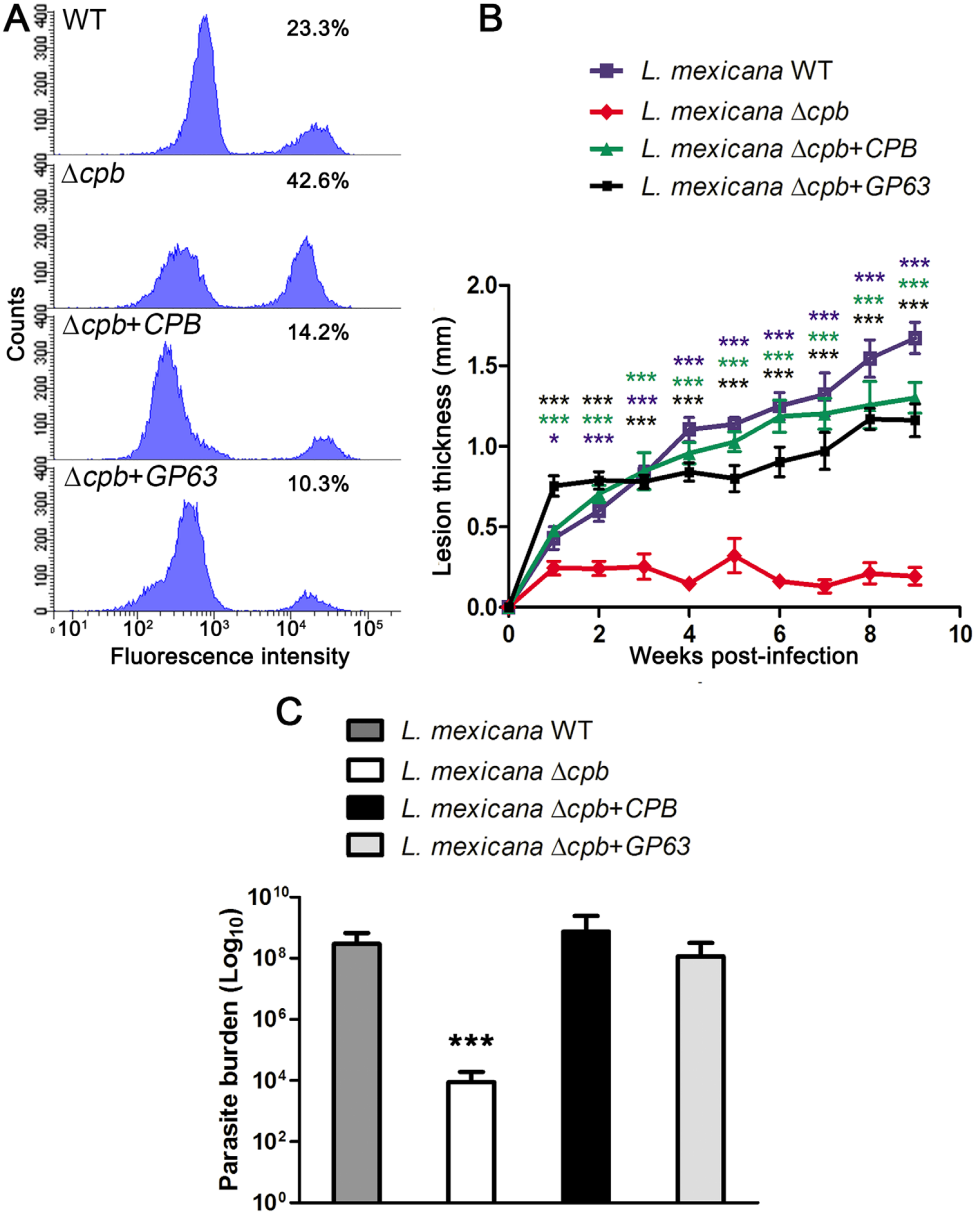
GP63 confers virulence to *L*. *mexicana* Δ*cpb*. Stationary phase *L*. *mexicana* (WT, Δ*cpb*, Δ*cpb*+*cpb* and Δ*cpb*+*GP63*) promastigotes were incubated in the presence of 20% human serum for 30 min, stained with a fixable viability dye, and then subjected to flow cytometry **(A)**. Mice were challenged with 5x10^6^ late-stationary phase *L*. *mexicana* (WT, Δ*cpb*, Δ*cpb*+*cpb* and Δ*cpb*+*GP63*) promastigotes that were injected subcutaneously into the hind footpad. Disease progression was monitored at weekly intervals, by measuring the thickness of the infected footpad and the contralateral uninfected footpad. **(B)**. Parasite burden was obtained by limiting dilution assay from infected homogenized footpads 9 weeks after inoculation **(C)**. Human serum lyses were performed in two independent experiments and six mice per group were used for the determination of lesion formation and parasite burden. Each data point represents the average ± SEM of 6 mice per group, and statistical significance was denoted by * p≤0.01, and *** p≤0.0001.

Since GP63 was shown to contribute to *L*. *major* virulence [32], we next sought to determine whether expression of GP63 is sufficient to restore the ability of Δ*cpb* to replicate inside macrophages and to cause lesions in mice [19]. To this end, we first infected BMM with either WT, Δ*cpb*, Δ*cpb*+*CPB*, or Δ*cpb*+*GP63* stationary phase promastigotes and we assessed parasite burden and PV surface area at various time points post-infection. We found that Δ*cpb* had an impaired capacity to replicate inside macrophages and to induce the formation of large communal PVs compared to WT and Δ*cpb*+*CPB* parasites (Fig 5A, 5B and 5C). Strikingly, expression of GP63 in Δ*cpb* restored its ability to replicate in macrophages and to induce large communal PVs up to 72 h post-infection. These results underline the role of GP63 in the ability of *L*. *mexicana* to infect and replicate in macrophages, even in the absence of CPB. Following inoculation inside the mammalian host, promastigotes are exposed to complement and both GP63 and LPG confer resistance to complement-mediated lysis [32, 33]. *L*. *mexicana* promastigotes were therefore analyzed for their sensitivity to complement-mediated lysis in the presence of fresh human serum. As shown in Fig 6A, over 40% of Δ*cpb* was killed after 30 min in the presence of 20% serum, whereas Δ*cpb*+*CPB*, and Δ*cpb*+*GP63* were more resistant to serum lysis at 14% and 10%, respectively. Absence of both GP63 and LPG may be responsible for the serum sensitivity of Δ*cpb*. Finally, to assess the impact of GP63 on the ability of Δ*cpb* to cause lesions, we used a mouse model of cutaneous leishmaniasis. Susceptible BALB/c mice were infected in the hind footpad with either WT, Δ*cpb*, Δ*cpb*+*CPB*, or Δ*cpb*+*GP63* promastigotes and disease progression was monitored for 9 weeks. Consistent with its reduced capacity to replicate inside macrophages, Δ*cpb* failed to cause significant lesions compared to WT parasites [19] and Δ*cpb* complemented with *CPB* (Fig 6B). Remarkably, expression of GP63 in Δ*cpb* restored its capacity to cause lesions, albeit to a lower level than Δ*cpb* complemented with *CPB*. Lesion size correlated with parasite burden, as measured at 9 weeks post-infection (Fig 6C). Collectively, these results indicate that expression of GP63 is sufficient to restore virulence of Δ*cpb*.

## Discussion

This study aimed at investigating the mechanism(s) by which CBP contributes to *L*. *mexicana* virulence. To this end, we initially examined PV biogenesis by assessing the impact of *L*. *mexicana* infection on the trafficking of VAMP3 and VAMP8, two endocytic SNAREs associated with phagosome biogenesis and function [1, 34]. We found that both SNAREs were down-modulated in a CPB-dependent manner, which hampered VAMP3 recruitment to PVs. We also discovered that expression of GP63, which we previously showed to be responsible for cleaving SNAREs in *L*. *major*-infected macrophages [1], was down-modulated in the *L*. *mexicana* Δ*cpb*. Strikingly, restoration of GP63 expression in Δ*cpb* bypassed the need for CPB for SNARE cleavage. Similarly, episomal expression of GP63 enabled the Δ*cpb* mutant to establish infection in macrophages, induce larger PVs and cause lesions in mice. These findings imply that CPB contributes to *L*. *mexicana* virulence in part through the regulation of GP63 expression, and provide evidence that GP63 is a key virulence factor for *L*. *mexicana*.

The observation that CPB regulates GP63 expression at the mRNA levels was both unexpected and intriguing. Insight into the possible mechanism(s) may be deduced from a recent study on the role of cathepsin B in *L*. *donovani*, which is homologous to the *L*. *mexicana* CPC [35]. Similar to *L*. *mexicana* Δ*cpb*, *L*. *donovani* Δ*catB* displays reduced virulence in macrophages. To investigate the role of cathepsin B in virulence, the authors performed quantitative proteome profiling of WT and Δ*catB* parasites and identified 83 proteins whose expression is altered in the absence of cathepsin B, with the majority being down-modulated [35]. Among those were a group of proteins involved in post-transcriptional regulation of gene expression (RNA stability, processing, translation) [35]. Whether this is the case in Δ*cpb* deserves further investigation. Clearly, a detailed analysis of wild-type and Δ*cpb* parasites may provide the information required to understand the extent of the impact of CBP on the expression and synthesis of virulence factors and the exact role of CPB in *L*. *mexicana* virulence. The observation that episomal expression of GP63 in Δ*cpb* restored LPG synthesis is an intriguing issue, as it suggests that GP63 acts on a LPG biosynthetic step. This role for GP63 is likely redundant, since *L*. *major* Δ*gp63* promastigotes express LPG levels similar to that of wild type parasites (S1 Fig).

It has been proposed that expansion of the PVs hosting parasites of the *L*. *mexicana* complex leads to the dilution of the microbicidal effectors to which the parasites are exposed, thereby contributing to parasite survival [36]. Both host and parasite factors may be involved in the control of PV enlargement. On the host side, it has been previously reported that *L*. *amazonensis* cannot survive in cells overexpressing *LYST*, a host gene that restricts *Leishmania* growth by counteracting PV expansion [37]. Similarly, disrupting the fusion between PVs housing *L*. *amazonensis* and the endoplasmic reticulum resulted in limited PV expansion and inhibition of parasite replication [15, 16]. On the parasite side, virulence of *L*. *amazonensis* isolates was shown to correlate with the ability to induce larger PVs [38]. Our results indicate that the inability of Δ*cpb* to multiply inside macrophages is associated with smaller PV size, and that expression of GP63 is sufficient to restore the capacity of Δ*cpb* to survive within macrophages and to induce PV expansion. How does GP63 modulate *L*. *mexicana* virulence and PV expansion? In addition to the numerous macrophage proteins known to be targeted by GP63, it is possible that SNARE cleavage is one of the factors associated with *L*. *mexicana* virulence and PV expansion. For instance, we previously reported that VAMP8 is required for phagosomal oxidative activity [1]. One may envision that its degradation by GP63 is part of a strategy used by *L*. *mexicana* to establish infection in an environment devoid of oxidants, thereby contributing to parasite survival. The LYST protein is a regulator of lysosome size and its absence leads to further PV expansion and enhanced *L*. *amazonensis* replication [37]. It is interesting to note that LYST was proposed to function as an adaptor protein that juxtaposes proteins such as SNAREs that mediate intracellular membrane fusion reactions [39]. In this context, cleavage of SNAREs that interact with LYST may interfere with its function and promote PV expansion. Further studies will be necessary to clarify these issues, including the potential role of VAMP3 and VAMP8 in PV biogenesis and expansion.

Previous studies using Δ*cpb* parasites led to the conclusion that CPB enables *L*. *mexicana* to alter host cell signaling and gene expression through the cleavage of various host proteins [20, 22]. Hence, CPB-dependent cleavage of PTP-1B, NF-κB, STAT1, and AP1 in *L*. *mexicana*-infected macrophages was associated with the inhibition of IL-12 expression and generation of nitric oxide, both of which are important for initiation of a host immune response and parasite killing, respectively. Our finding that GP63 expression is down-modulated in the Δ*cpb* mutant raises the possibility that cleavage of those transcription factors may actually be mediated by GP63. Indeed, GP63 cleaves numerous host macrophage effectors, including PTP-1B, NF-κB, STAT1, and AP1 [40]. Revisiting the role of CPB in the context of GP63 expression will be necessary to elucidate whether, and to which extent, CPB is acting directly on the host cell proteome.

In sum, we discovered that CPB contributes to *L*. *mexicana* virulence in part through the regulation of GP63 expression. Complementation of *Δcpb* revealed the importance of GP63 for the virulence of *L*. *mexicana*, as it participates in the survival of intracellular parasites, PV expansion, and the formation of cutaneous lesions.

## Materials and Methods

### Ethics statement

Experiments involving mice were done as prescribed by protocol 1406–02, which was approved by the *Comité Institutionnel de Protection des Animaux* of the INRS-Institut Armand-Frappier. *In vivo* infections were performed as per Animal Use Protocol #4859, which was approved by the Institutional Animal Care and Use Committees at McGill University. These protocols respect procedures on good animal practice provided by the Canadian Council on Animal Care (CCAC).

### Antibodies

The mouse anti-GP63 monoclonal antibody was previously described [41]. The mouse anti-phosphoglycans CA7AE monoclonal antibody [42] was from Cedarlane and the rabbit polyclonal anti-aldolase was a gift from Dr. A. Jardim (McGill University). Rabbit polyclonal antibodies for VAMP3 and VAMP8 were obtained from Synaptic Systems.

### Cell culture

Bone marrow-derived macrophages (BMM) were differentiated from the bone marrow of 6- to 8-week-old female 129XB6 mice (Charles River Laboratories) as previously described [43]. Cells were cultured for 7 days in complete medium (DMEM [Life Technologies] supplemented with L-glutamine [Life Technologies], 10% heat-inactivated FBS [PAA Laboratories], 10 mM HEPES at pH 7.4, and antibiotics) containing 15% v/v L929 cell–conditioned medium as a source of M-CSF. Macrophages were kept at 37°C in a humidified incubator with 5% CO_2_. To render BMM quiescent prior to experiments, cells were transferred to 6- or 24-well tissue culture microplates (TrueLine) and kept for 16 h in complete DMEM without L929 cell–conditioned medium. Promastigotes of *L*. *mexicana* wild-type strain (MNYC/BZ/62/M379) and of *L*. *major* NIH S (MHOM/SN/74/Seidman) clone A2 were grown at 26°C in *Leishmania* medium (Medium 199 supplemented with 10% heat-inactivated FBS, 40 mM HEPES pH 7.4, 100 μM hypoxanthine, 5 μM hemin, 3 μM biopterin, 1 μM biotin, and antibiotics). The isogenic *L*. *mexicana* CPB-deficient mutant Δ*cpb* ^*pac*^ (thereafter referred to as Δ*cpb*) and its complemented counterpart Δ*cpb* ^*pac*^[pGL263] (thereafter referred to as Δ*cpb+CPB)* were described previously [21]. *L*. *mexicana* Δ*cpb* promastigotes were electroporated as described [44] with the pLEXNeoGP63.1 plasmid [32] to generate Δ*cpb+GP63* parasites. *L*. *mexicana* Δ*cpb+CPB* and Δ*cpb+GP63* promastigotes were grown in the presence of 50 μg/ml hygromycin or 50 μg/ml G418, respectively. The *L*. *major* NIH clone A2 isogenic Δ*gp63* mutant and its complemented counterpart Δ*gp63*+*gp63* have been previously described [32]. Cultures of Δ*gp63*+*gp63* promastigotes were supplemented with 50 μg/ml G418.

### Synchronized phagocytosis assays

Prior to macrophage infections, promastigotes in late stationary phase were opsonized with DBA/2 mouse serum. For synchronized phagocytosis using parasites, macrophages and parasites were incubated at 4°C for 10 min and spun at 167 *g* for 1 min, and internalization was triggered by transferring cells to 34°C. Macrophages were washed twice after 2h with complete DMEM to remove the non-internalized parasites and were further incubated at 34°C for the required times. Cells were then washed with PBS and stained using the Hema 3 Fixative and Solutions kit (Fisher HealthCare), or prepared for confocal immunofluorescence microscopy.

### Confocal immunofluorescence microscopy

Macrophages on coverslips were fixed with 2% paraformaldehyde (Canemco and Mirvac) for 40 min and blocked/permeabilized for 17 min with a solution of 0.05% saponin, 1% BSA, 6% skim milk, 2% goat serum, and 50% FBS. This was followed by a 2 h incubation with primary antibodies diluted in PBS. Macrophages were then incubated with a suitable combination of secondary antibodies (anti-rabbit Alexa Fluor 488 and anti-rat 568; Molecular Probes) and DAPI (Life technologies). Coverslips were washed three times with PBS after every step. After the final washes, Fluoromount-G (Southern Biotechnology Associates) was used to mount coverslips on glass slides, and coverslips were sealed with nail polish (Sally Hansen). Macrophages were imaged with the 63X objective of an LSM780 microscope (Carl Zeiss Microimaging), and images were taken in sequential scanning mode. Image analysis and vacuole size measurements were performed with the ZEN 2012 software.

### Electrophoresis, western blotting, and zymography

Prior to lysis, macrophages were placed on ice and washed with PBS containing 1 mM sodium orthovanadate and 5 mM 1,10-phenanthroline (Roche). Cells were scraped in the presence of lysis buffer containing 1% Nonidet P-40 (Caledon), 50 mM Tris-HCl (pH 7.5) (Bioshop), 150 mM NaCl, 1 mM EDTA (pH 8), 5 mM 1,10-phenanthroline, and phosphatase and protease inhibitors (Roche). Parasites were washed twice with PBS and lysed in the presence of lysis buffer containing 0.5% Nonidet P-40 (Caledon), 100mM Tris-HCl (Bioshop) and 150 mM NaCl at -70°C. Lysates were thawed on ice and centrifuged for 10 min to remove insoluble matter. After protein quantification, protein samples were boiled (100°C) for 6 min in SDS sample buffer and migrated in 12% or 15% SDS-PAGE gels. Three micrograms and 15 μg of protein were loaded for parasite and infected macrophage lysates, respectively. Proteins were transferred onto Hybond-ECL membranes (Amersham Biosciences), blocked for 1 h in TBS 1X-0.1% Tween containing 5% skim milk, incubated with primary antibodies (diluted in TBS 1X-0.1% Tween containing 5% BSA) overnight at 4°C, and thence with appropriate HRP-conjugated secondary antibodies for 1 h at room temperature. Then, membranes were incubated in ECL (GE Healthcare), and immunodetection was achieved via chemiluminescence. Membranes were washed 3 times between each step. For zymography, 2 μg of lysate were incubated at RT for 6 min in sample buffer without DTT and then migrated in 12% SDS-PAGE gels with 0.2% gelatin (Sigma). Gels were incubated for 1 h in the presence of 50 mM Tris pH 7.4, 2,5% Triton X-100, 5 mM CaCl_2_ and 1 μM ZnCl_2_ and incubated overnight in the presence of 50 mM Tris pH 7.4, 5 mM CaCl_2_, 1μM ZnCl_2_ and 0,01% NaN_3_ at 37°C [45].

### FACS analysis

Late stationary phase promastigotes were incubated for 30 min in complete DMEM medium with 20% human serum from healthy donors. Parasites were then incubated in LIVE/DEAD Fixable Violet Dead Cell Stain Kit (Life technologies) and fixed in 2% paraformaldehyde. Flow cytometric analysis was carried out using the LSRFortessa cytometer (Special Order Research Product; BD Biosciences), and the BD FACSDiva Software (version 6.2) was used for data acquisition and analysis.

### Mouse infections

Male BALB/c mice (6 to 8 weeks old) were purchased from Charles River Laboratories and infected in the right hind footpad with 5x10^6^ stationary phase *L*. *mexicana* promastigotes as described [46]. Disease progression was monitored by measuring footpad swelling weekly with a metric caliper, for up to 9 weeks post-infection. Footpads were then processed to calculate parasite burden using the limiting dilution assay.

### Limiting dilution assay

After 9 weeks of infection, mice were euthanized under CO_2_ asphyxiation and subsequently by cervical dislocation. The infected footpads were surface-sterilized with a chlorine dioxide based disinfectant followed by ethanol 70% for 5 min. Footpads were washed in PBS, lightly sliced, transferred to a glass tissue homogenizer containing 6 ml of PBS, and manually homogenized. The last step was repeated two to three times, until complete tissue disruption was achieved. Final homogenate was then centrifuged at 3,000 x *g* for 5 min and resuspended in the appropriate volume of PBS. 100 μl of homogenate were added in duplicates to 96-well plates containing 100 μl of complete Schneider’s medium in each well (twenty-four 2-fold dilutions for each duplicate). Plates were incubated at 28°C. After 7–10 days, the number of viable parasites was determined from the highest dilutions at which promastigotes were observed using an inverted microscope [47].

### Reverse transcription-PCR (RT-PCR)

Total RNA was extracted from promastigotes using the TRIzol reagent (Invitrogen Life Technology, Carlsbad, CA) and reverse transcribed. One-tenth of the resulting cDNA was amplified by PCR on a DNA thermal cycler, version 2.3 (Perkin-Elmer Corporation, Norwalk, CT), with the following primer pairs: for the *L*. *mexicana GP63* C-1 5'-ACCGTCTGAGAGTCGGAACT-3' (forward), 5'-GTAGTCCAGGAATGGCGAGT-3' (reverse); the *L*. *major GP63-1* 5'-TCTGAGGCACATGCTTCGTT-3' (forward), 5'-GTCAGTTGCCTTCGGTCTGA-3' (reverse), the *L*. *mexicana LPG2* 5'CATTTGGTATCCTGGTGCTG-3' (forward), 5'-GAGGAAGCCACTGTTAGCC-3' (reverse), and the *L*. *mexicana* α-tubulin 5'-CTATCTGCATCCACATTGGC-3' (forward), 5'-ACTTGTCAGAGGGCATGGA-3' (reverse). The PCR products were analyzed by electrophoresis on a 3% (wt/vol) agarose gel, and the pictures were taken using AlphaImager 3400 imaging software (Alpha Innotech Corporation, San Leandro, CA).

### Statistical analyses

Statistically significant differences were analyzed by ANOVA followed by the Tukey post-hoc test using the Graphpad Prism program (version 5.0). For the limiting dilution assay, the non-parametric Mann-Whitney or Kruskal-Wallis test was used. Values starting at P<0.05 were considered statistically significant. All data are presented as mean ± SEM.

## Supporting Information

S1 Fig *L*. *major* Δ*gp63* promastigotes express normal levels of LPG. Stationary phase promastigotes were lysed and total cell extracts were analysed by Western blotting for LPG levels. Similar results were obtained in two separate experiments.
(TIF)
