# Supplementary figures and images for: Cysteine Peptidase B Regulates Leishmania mexicana Virulence through the Modulation of GP63 Expression
Source: PLoS Pathog. 2016 May 18;12(5):e1005658. doi: 10.1371/journal.ppat.1005658 (PMC4871588; doi:10.1371/journal.ppat.1005658)

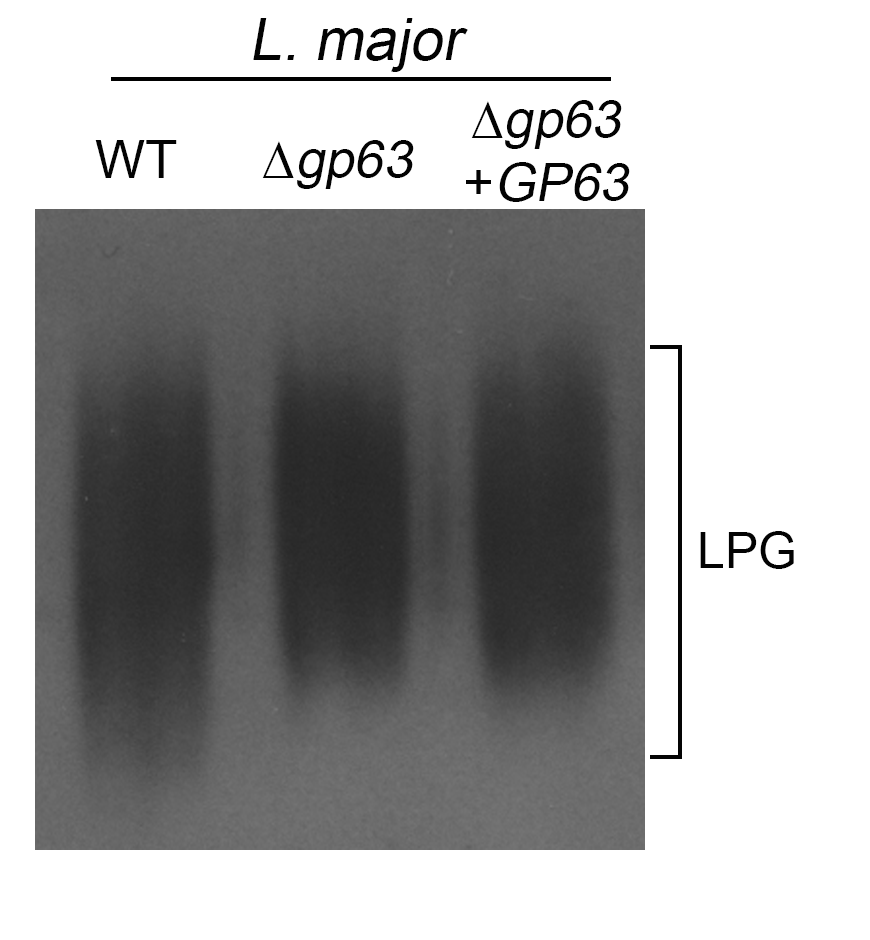

Supplement: S1 Fig — Stationary phase promastigotes were lysed and total cell extracts were analysed by Western blotting for LPG levels. Similar results were obtained in two separate experiments. (TIF) [file ppat.1005658.s001.tif]
